# Remarkable Improvements After Cataract Surgery in a Presumed “End-Stage” Glaucoma Patient: A Case Report

**DOI:** 10.7759/cureus.44683

**Published:** 2023-09-04

**Authors:** Hani El Helwe, Zoë Ingram, Daniel Liebman, Henisk Falah, David A Solá-Del Valle

**Affiliations:** 1 Ophthalmology, Harvard Medical School, Boston, USA; 2 Glaucoma, Massachusetts Eye and Ear, Boston, USA

**Keywords:** continuous wave, micropulse, diode laser, transscleral cyclophotocoagulation, glaucoma, cataract surgery

## Abstract

Predicting the visual outcome after cataract extraction can be challenging in glaucoma patients who develop cataracts. Here, we demonstrate the case of a patient with advanced glaucoma and a mild-to-moderate cataract at initial presentation, who demonstrated remarkable improvement in visual acuity following a period of controlled intraocular pressure (IOP) and the removal of a matured cataract at the time of surgery. A 64-year-old Haitian woman with severe mixed-mechanism glaucoma and hand motion vision in both eyes (OU) presented with intraocular pressures of 38 mmHg OD (oculus dexter/right eye) and 41 mmHg OS (oculus sinister/left eye) while on three IOP-lowering agents. Her medications were escalated to six IOP-lowering medications, and she underwent bilateral transscleral laser cyclophotocoagulation with both micropulse and continuous wave probes simultaneously. Postoperatively, IOPs dropped to 7 and 9 mmHg in the right and left eyes, respectively, and remained at or below target on three topical agents for the remainder of her follow-up. Contrastingly, the patient's cataract had progressed, and the decision was made to undergo cataract extraction OU sequentially. The subsequent clinical course demonstrated progressive visual improvement with 20/80 best-corrected visual acuity OU and increased independence with activities of daily living. This case illustrates the potential for visual improvement in an advanced glaucoma patient after removing a matured cataract despite limited prior expectations. Ocular comorbidities complicate but do not necessarily preclude appropriate interventions that may improve patients' vision-related quality of life.

## Introduction

Cataracts and glaucoma are the top two leading causes of blindness in individuals above 50 years, with a global prevalence of 15.2 and 3.6 million cases, respectively, in 2020 [1]. As the prevalence of these two diseases increases with age, it is not uncommon to find them coexisting together in a single individual [2-5]. Some studies have even suggested that glaucoma is a risk factor for cataract development and progression [6-8]. When patients have both glaucoma and cataracts, it can be challenging to elucidate which diagnosis is the main contributor to their visual impairment. Additionally, predicting the visual potential after cataract extraction can be challenging in these patients. Here, we demonstrate the case of a patient with advanced glaucoma and a mild-to-moderate cataract at initial presentation, who demonstrated remarkable improvement in visual acuity following a period of controlled intraocular pressure (IOP) and the removal of a matured cataract at the time of surgery.

## Case presentation

Clinical history and acute treatment

A 65-year-old Haitian woman presented to the Emergency Department complaining of gradually worsening vision in both eyes (OU) in April 2019. She was previously diagnosed with severe mixed-mechanism glaucoma OU and treated with an unknown laser procedure in addition to a medication regimen consisting of brimonidine thrice daily OU, latanoprost every night OU, and acetazolamide orally (unclear dose). The patient reported nonadherence to her glaucoma medications after a recent trip to Haiti two weeks prior.

On presentation, her visual acuity was hand motion (HM) in both eyes, and her IOP was 26 mmHg in her right eye (OD) and 38 mmHg in her left eye (OS) using Tono-Pen AVIA® Handheld Tonometer (Reichert, Inc., New York, United States). Her slit lamp exam showed mild-to-moderate cataracts OU with an unmistakably clear view of the fundus and near total cupping of optic nerves OU. Corneas were clear. She had open angles with peripheral anterior synechia (PAS) in both eyes on gonioscopy. After three rounds of topical dorzolamide, brimonidine, latanoprost, timolol, and oral acetazolamide, her IOPs dropped to 19 mmHg OD and 21 mmHg OS, and a glaucoma referral was placed.

**Figure 1:**
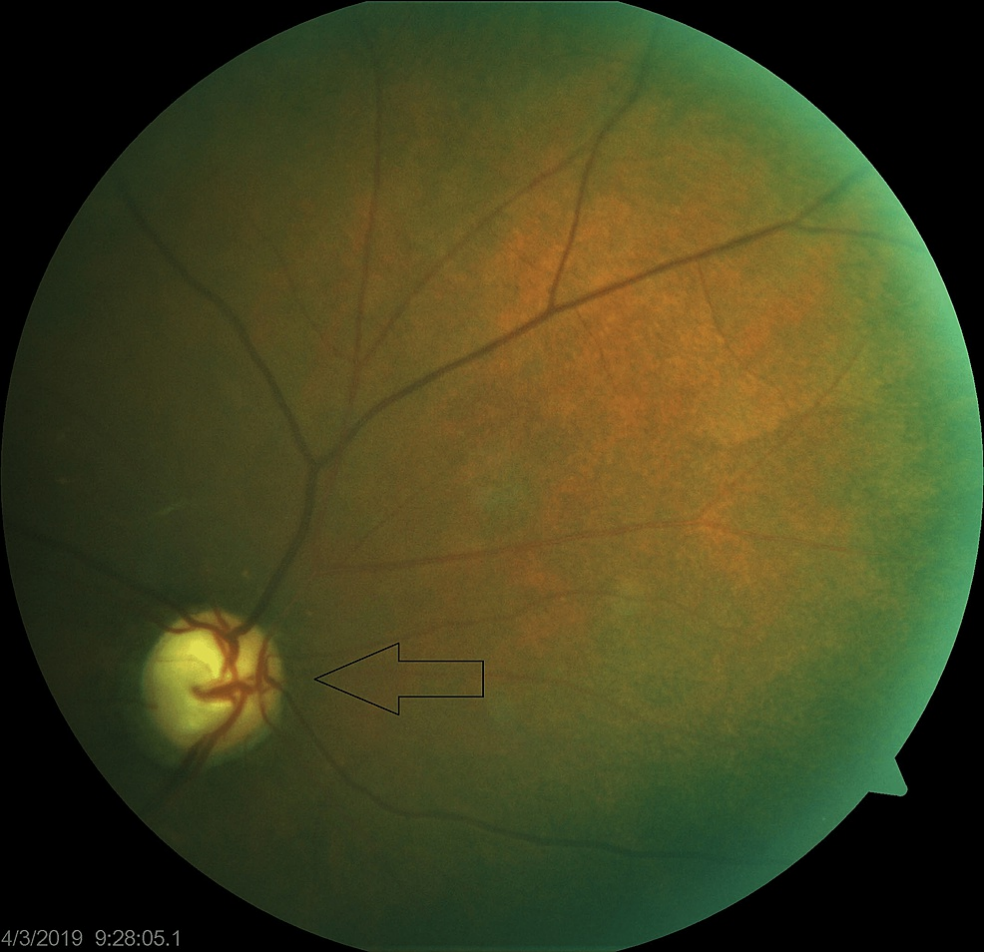
Optic Nerve Head (ONH) of Right Eye (April 3, 2019)

**Figure 2:**
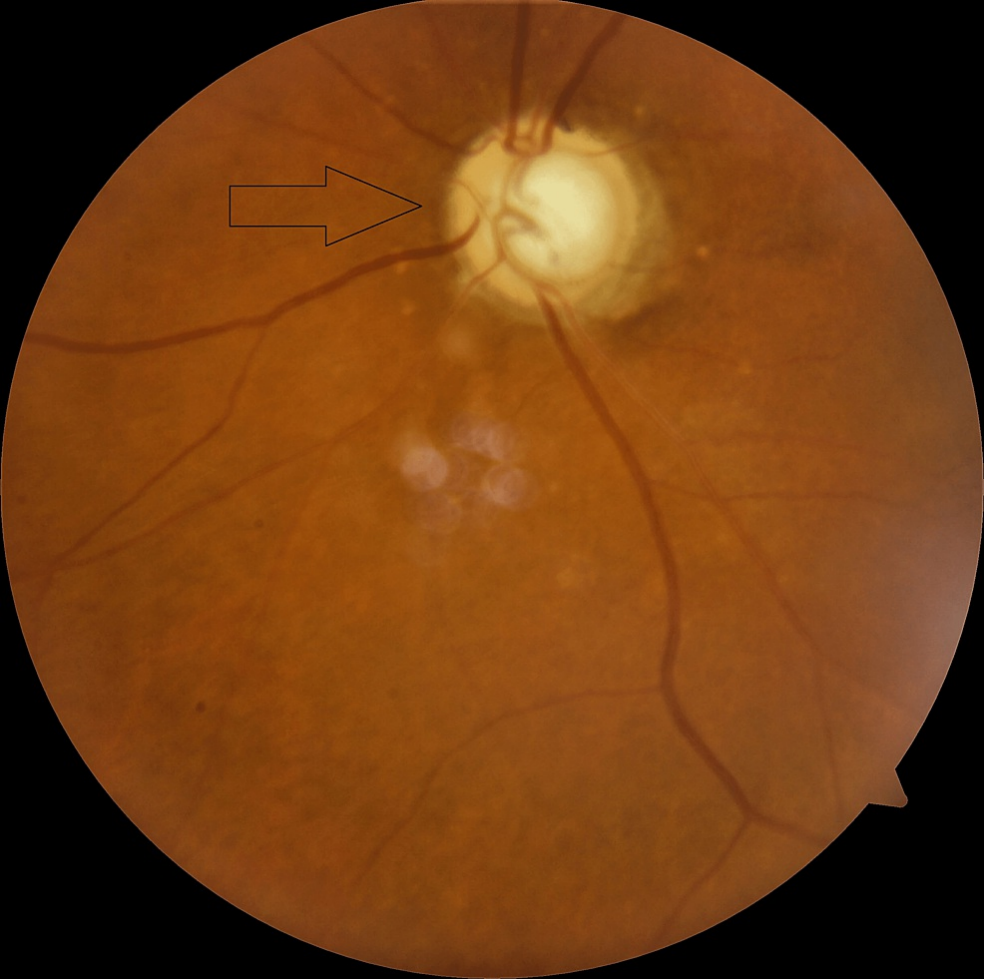
Optic Nerve Head (ONH) of Left Eye (April 3, 2019)

The patient presented to the glaucoma clinic at three months later, complaining of inability to perform activities of daily living (ADLs) without assistance. She denied any ocular pain. Her applanation tonometry pressures were 38 mmHg OD and 41 mmHg OS while on timolol twice daily OU, brimonidine thrice daily OU, and acetazolamide 500 mg orally twice daily. The patient had a best corrected visual acuity (BCVA) of HM OU. Slit lamp exam was relevant for 1-2+ nuclear sclerosis with trace cortical spokes OU. Clear corneas were observed without any evidence of microcystic edema. No conjunctival injection or evidence of prior laser peripheral iridotomy (LPI) was observed. Pachymetry showed thin corneas, averaging 478 μm OD and 473 μm OS. Gonioscopy showed 2+ pigmented trabecular meshwork where visible, prominent iris processes with superior, temporal, and inferior PAS OD, and 360° scattered PAS OS. Dilated fundus exam revealed cup-to-disc ratios (CDR) of 0.95 OD and 0.99 OS with peripapillary atrophy and diffuse thinning in both eyes (Figure 1 and Figure 2).

**Figure 3:**
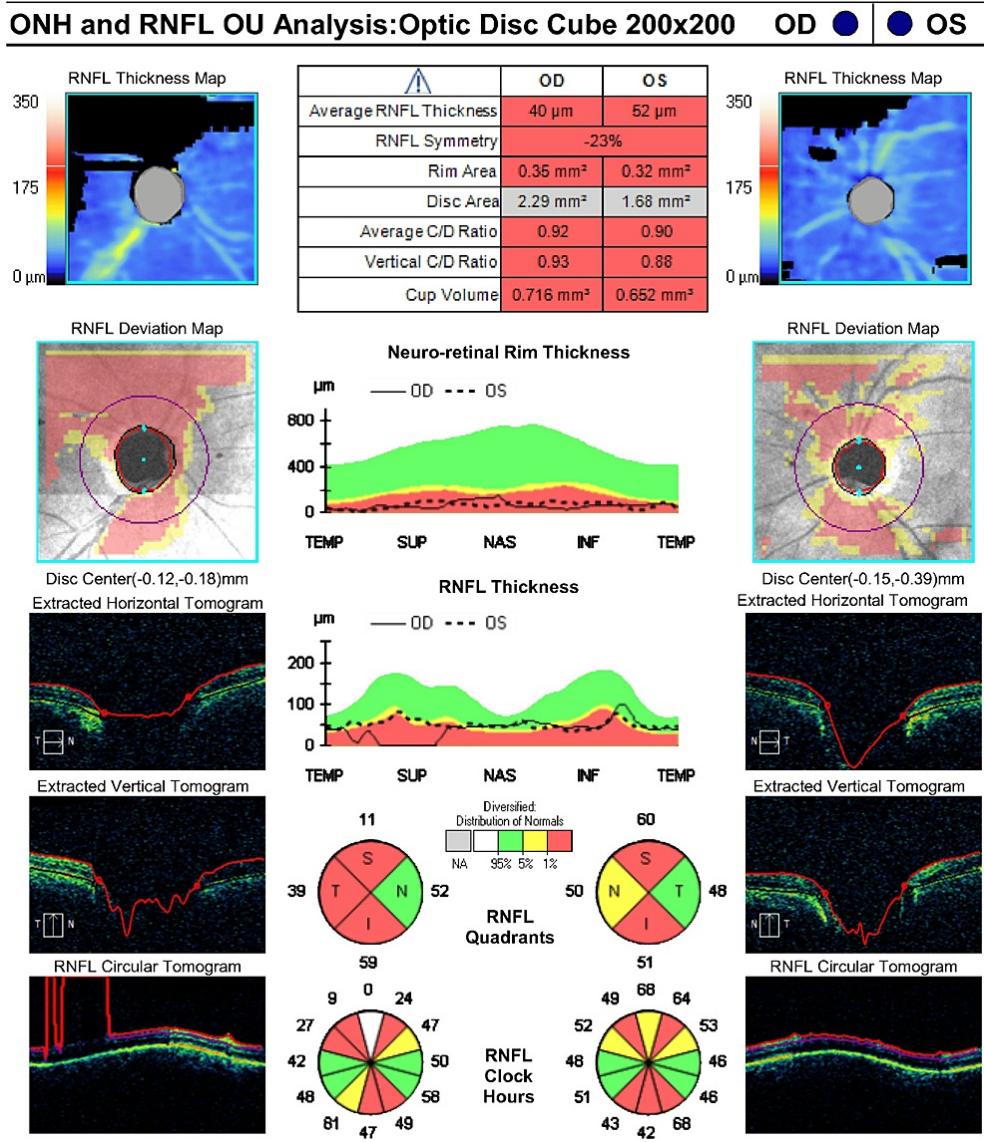
Optical Coherence Tomography (OCT) Optic Nerve Head (ONH) and Retinal Nerve Fiber Layer (RNFL) Analysis of Both Eyes (April 3, 2019)

**Figure 4:**
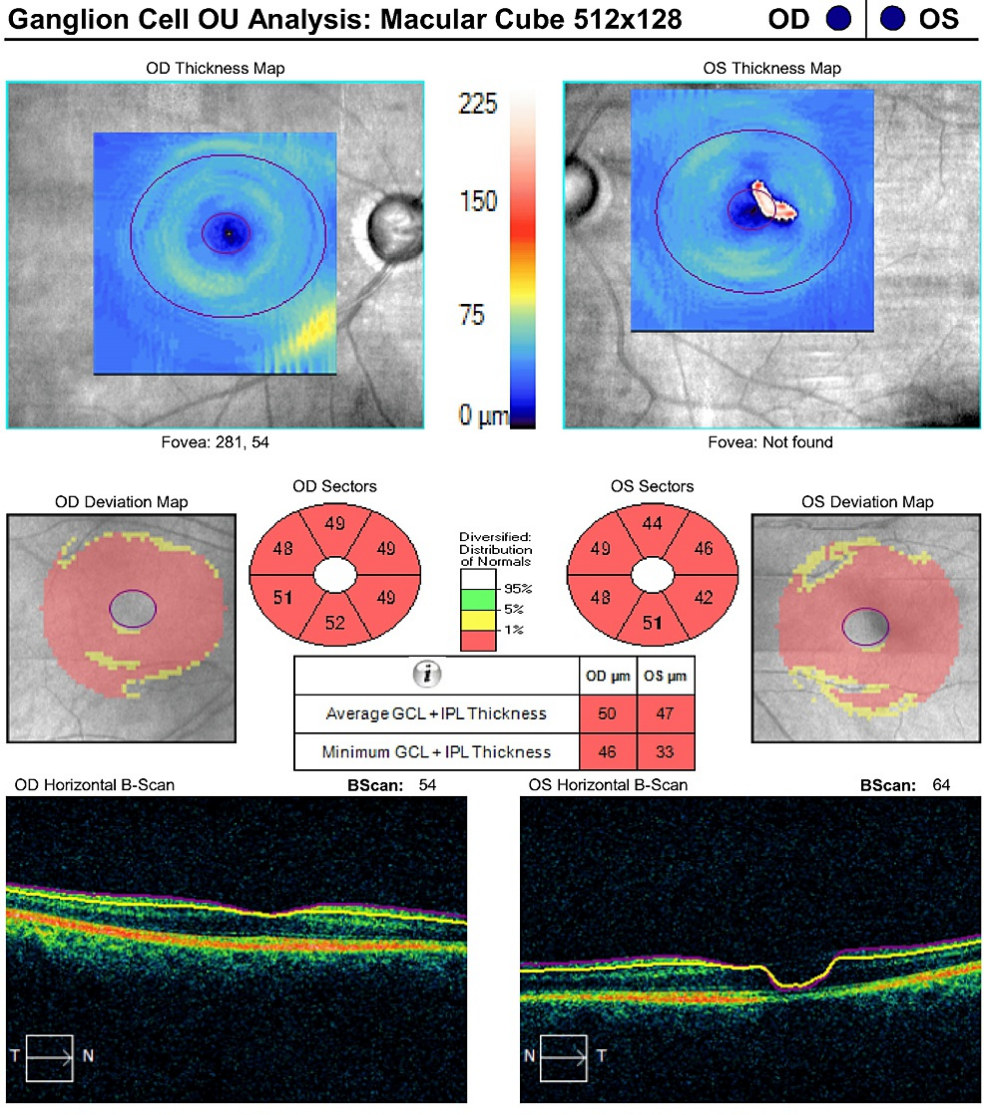
Optical Coherence Tomography (OCT) Ganglion Cell Layer (GCL) Analysis of Both Eyes (April 3, 2019)

This finding was confirmed by optical coherence tomography (OCT) with retinal nerve fiber layer (RNFL) measurements that were at floor, although the quality of the OCT was subpar due to eyelid artifacts (Figure 3 and Figure 4). No disc hemorrhages were observed. Functional testing with Humphrey visual fields (HVF) was deferred due to HM vision OU. Her exam was consistent with advanced mixed-mechanism (open-angle glaucoma (OAG)/PAS) OU. She had several glaucoma risk factors, including age above 60, thin corneas, Afro-Caribbean ethnicity, and a family history of glaucoma in her brother. Her vascular and perfusion risk factors included type 2 diabetes mellitus, hypertension, and dyslipidemia treated with dietary modification, metoprolol orally once daily, lisinopril orally once daily, and atorvastatin orally once daily. She denied any history of asthma, long-term systemic steroid use, trauma, or kidney disease. A target IOP of 12 mmHg OU was set given her presumed end-stage glaucoma with thin corneas, and the patient’s treatment regimen was escalated to timolol twice daily OU, latanoprost at night OU, brimonidine thrice daily OU, dorzolamide thrice daily OU, netarsudil at night OU, and acetazolamide 500 mg PO thrice daily (from twice daily). Given that her IOP was well above goal, the risks and benefits of various laser and surgical interventions were weighed. The patient and surgeon agreed to proceed with transscleral laser cyclophotocoagulation (TSCPC) with both micropulse (MP) and continuous wave (CW) simultaneously OU, a combination procedure previously described by Nirappel et al. [9].

Augmented micropulse cyclophotocoagulation with limited G-probe application

The patient was brought to the operating room, where general anesthesia was induced, and a time-out procedure was performed. General anesthesia was selected to avoid any blood pressure or heart rhythm spikes that could interfere with the patient's surgery, given her history of anxiety, uncontrolled hypertension, and sinus tachycardia. We wanted to avoid simultaneous peribulbar eye blocks and allow for faster recovery. The patient's operative eye and ocular adnexa were then sterilized with a 5% Betadine solution. Attention was first given to the right eye. Micropulse transscleral cyclophotocoagulation (MP-TSCPC) was performed with MicroPulse P3® (IRIDEX Corporation, Mountain View, California, United States), followed by continuous-wave transscleral cyclophotocoagulation (CW-TSCPC) with G-Probe® Delivery Device (IRIDEX Corporation). The IRIDEX 810-nm laser device, Cyclo G6® Glaucoma Laser System (IRIDEX Corporation) was used with both probes. Augmented MP-TSCPC was performed as previously described by Nirappel et al. [9]. Two 180-second applications, one for each hemisphere, were employed. Treatment settings were set at 2200 mW power and 31.3% duty cycles. Each 180-second application was split into a 90-second “sweeping” technique and a 90-second “stop-and-continue” technique. Following augmented MP-TSCPC, a limited procedure with CW-TSCPC was performed with the G-probe, as previously described by Gupta et al. [10]. Five 800-1600 mW spots were applied to each hemisphere using the G-probe. The 3 and 9 o'clock meridians were avoided throughout the procedure to minimize damage to the long posterior vessels and nerves. The same procedure was repeated in the left eye. Immediately after the procedure, the patient received a subconjunctival injection of 1-2 mL of 0.5 mg dexamethasone with antibiotics in each eye. Maxitrol ointment was placed on each eye before they were patched and shielded. Relevant visit data for both eyes are provided in Table 1.

**Table 1:** Summary of Patient Visit Data of Both Eyes from April 2019 to June 2022. OD = right; OS = left eye; OU = both eyes; IOP = intraocular pressure; TSCPC = transscleral cyclophotocoagulation; POD = postoperative day; POW = postoperative week; POM = postoperative month; NS = nuclear sclerosis; CS = cortical spokes; PCIOL = posterior chamber intraocular lens; BCVA = best corrected visual acuity; HM = hand motion; LP = light perception; Phaco = phacoemulsification

Visit Type	Visit Date	IOP OD/OS (mmHg)	BCVA OD	BCVA OS	Lens	Fundus View
Preoperative	April 3, 2019	38/41	HM	HM	1+ NS, CS OD; 1+ NS, CS OS	Clear OU
POD 1, TSCPC OU	April 16, 2019	20/25	LP	HM	1+ NS, CS OD; 1+ NS, CS OS	Clear OU
POW 5	May 23, 2019	07/09	LP	HM	1-2+ NS, CS OD; 1+ NS, CS OS	Clear OU
POM 3	July 18, 2019	09/17	HM	HM	1-2+ NS, CS OD; 1-2+ NS, CS OS	Clear OU
POM 5	September 26, 2019	06/10	HM	HM	2+ NS, CS OD; 1-2+ NS, CS OS	Hazy OD; Clear OS
POM 7	January 30, 2020	09/12	HM	HM	2+ NS, CS OD; 1-2+ NS, CS OS	Hazy OD; Clear OS
POM 11	May 21, 2020	07/17	LP	HM	White cataract OD; 2+ NS, CS OS	No View OD; Hazy OS
POM 15	September 3, 2020	08/11	LP	HM	White cataract OD; 2+ NS, CS OS	No View OD; Hazy OS
POM 18	December 3, 2020	07/12	LP	LP	White cataract OD; 2+ NS, CS OS	No View OD; Hazy OS
POM 20	February 4, 2021	07/11	LP	LP	White cataract OD; 2+ NS, CS OS	No View OD; Hazy OS
POD 1, Phaco OD	March 9, 2021	11/11	HM	LP	PCIOL OD; White cataract OS	No View OD; Hazy OS
POW 6	May 20, 2021	07/12	20/300	LP	PCIOL OD; White cataract OS	Hazy OU
POD 1, Phaco OS	August 26, 2021	10/13	20/300	20/400	PCIOL OD; PCIOL OS	Clear OD, Hazy OS
POW 6	November 16, 2021	07/10	20/250	20/100	PCIOL OD; PCIOL OS	Clear OU
Vision Rehab Clinic	June 17, 2022		20/80	20/80		

IOP control and remarkable cataract surgery outcome

**Figure 5:**
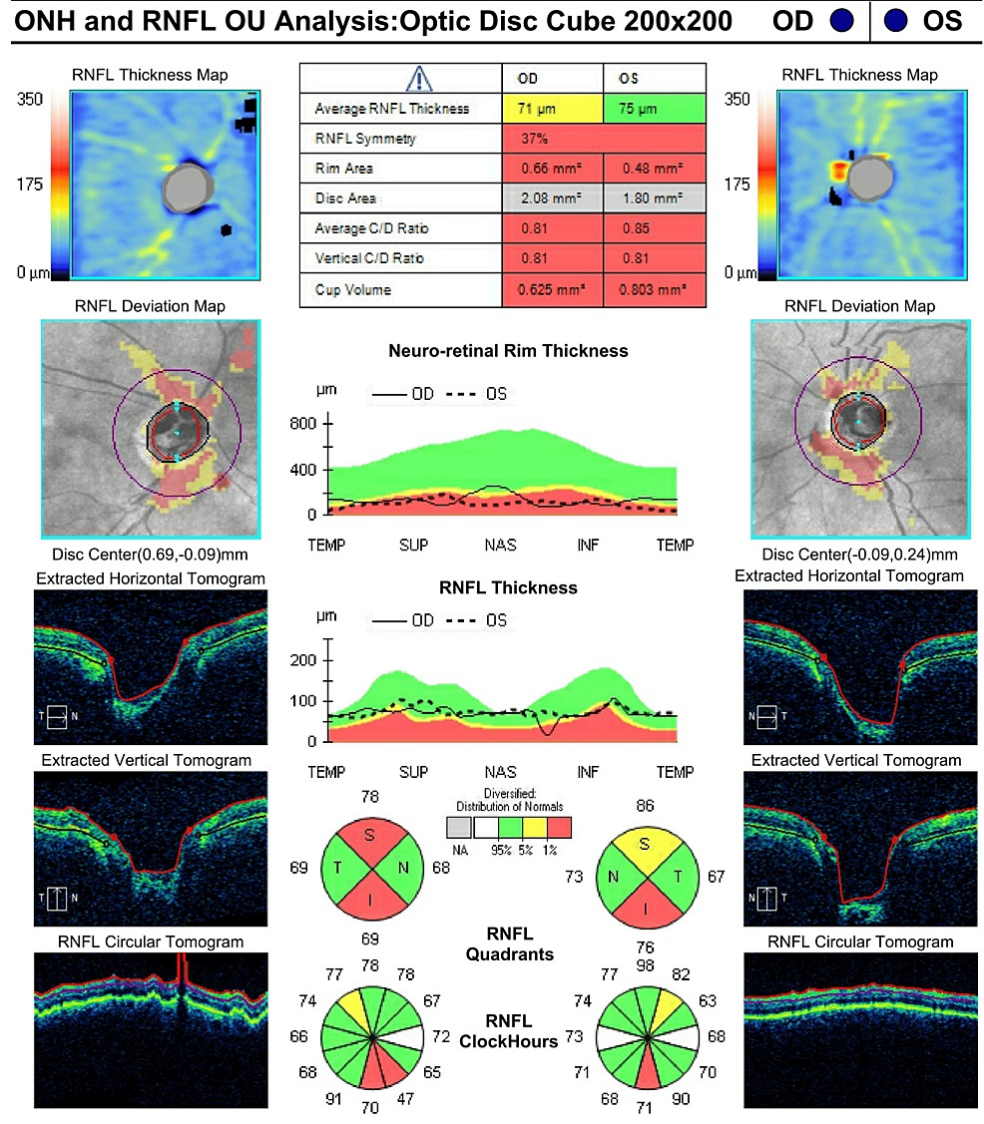
Optical Coherence Tomography (OCT) Optic Nerve Head (ONH) and Retinal Nerve Fiber Layer (RNFL) Analysis of Both Eyes in November 2021.

**Figure 6:**
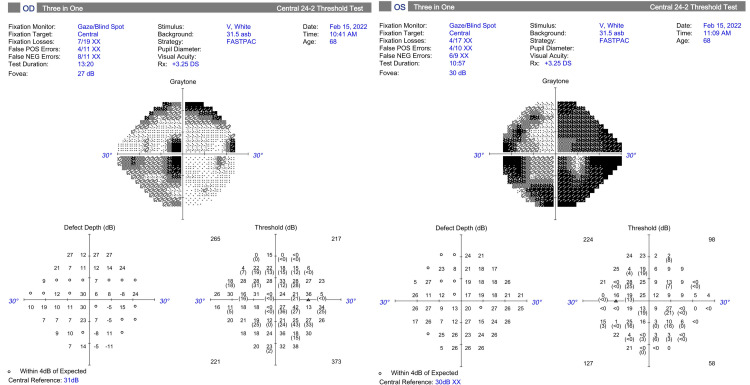
Humphrey Visual Field (HVF) Analysis in February 2022 of Both Eyes (February 15, 2022).

On postoperative day 1, the IOP measured 20 mmHg OD and 25 mmHg OS. Dorzolamide thrice daily OU and acetazolamide 500 mg orally thrice daily were withheld, and the patient was started on prednisolone acetate 1% four times a day OU, which was slowly tapered over 10 weeks. By postoperative week 5, her IOP was in the single digits at 7 mmHg OD and 9 mmHg OS, and her IOP-lowering medications were reduced to timolol twice daily OU, latanoprost at night OU, and brimonidine twice daily OU. Over the next 17 months, her glaucoma remained stable, and IOP OD remained at or below target pressure throughout the follow-up period. Contrastingly, the patient's cataract had progressed, with a mature, white cataract OD and 2+ NS with 2-3+ cortical spokes OS observed at POM 20, rendering any attempt to evaluate the posterior pole via slit lamp or OCT no longer possible. At this point, the patient was light perception OU and complained of deteriorating vision OD. A lengthy discussion with the patient explaining the risks and benefits of cataract surgery, in addition to the options of concomitant glaucoma surgery, was initiated. Considering the patient’s white cataract and advanced glaucoma status, the increased risk for complications and the likely limited visual potential after surgery were emphasized. The patient and surgeon agreed to proceed with standalone phacoemulsification with posterior chamber intraocular lens implantation (phaco), beginning with the right eye and following up with the left eye after six weeks. The subsequent clinical course demonstrated progressive visual improvement and increased independence with ADLs. BCVA was 20/80 in both eyes together during a vision rehab clinic visit 10 months after phaco OS, a remarkable improvement in visual function after phaco OU in this advanced glaucoma patient. IOP remained stable in the single-digit to low-teen range on two agents OD and two to four agents OS. Posterior pole evaluation via slit-lamp and OCT was rendered once again possible. RNFL thickness was measured to be 71 μm OD and 75 μm OS (Figure 5). A 24-2 HVF with size V stimuli was acquired for the first time and demonstrated severe visual loss but with remaining central islands of vision OU (Figure 6). 

## Discussion

Elderly individuals with refractory glaucoma and limited visual potential

Our 65-year-old female patient had optic nerve abnormalities consistent with advanced glaucoma, HM vision, and very high IOPs on maximum-tolerated medical therapy (MTMT), so she fell under the category of elderly individuals with limited visual potential. These individuals constitute a subset of glaucoma patients for whom management is exceptionally challenging. They often have other ocular pathologies in combination with glaucoma. A cataractous lens, for instance, can become intumescent, and glaucoma progression detected on OCT can be confounded by cataracts, which can artificially or non-artificially alter retinal thickness measurements [11]. As the cataract progresses, the view of the fundus becomes hazy, and estimating the CDR used in glaucoma follow-up becomes unreliable. The requisition of a standardized automated perimetry test with both size III and size V stimuli requires a minimum amount of visual acuity. It is thus rendered ineffective in patients with poor vision. These elderly individuals often have advanced visual field defects, and it is not uncommon for them to see through a small central island of functional vision.

Laser cyclophotocoagulation

The best next step in management is based on eye-specific and patient-specific characteristics. Incisional ocular surgeries have many known complications that may occur in any glaucoma patient, but the “Wipe-out” or “Snuff-out” phenomenon is typically described in older patients with advanced visual field loss affecting the central field and high baseline IOP despite medical therapy [12]. The phenomenon refers to an irreversible loss of central vision after glaucoma surgery in patients with advanced disease, with a reported incidence of 0-13.6% [13-17].

Diode laser cyclophotocoagulation has been historically reserved for eyes with advanced glaucomatous neuropathy with poor visual acuity and uncontrolled IOP. These eyes are at higher risk of nerve fiber damage from the sudden decompression inherent to procedures such as trabeculectomy or tube insertion in which an opening is created. Cases at high risk of failure or with already proven failure of incisional filtering surgery or glaucoma drainage device (GDDs) insertion are also more suitable for diode laser cyclophotocoagulation. Patients in whom there is a concern about ensuring strict follow-up after surgery are also candidates for diode lasers (American Academy of Ophthalmology recommendations, 2010) owing to its limited postoperative follow-up requirement [18].

Cyclodestructive procedures have been a vital treatment choice since the 1930s, with the most recent introduction in 1992 of a solid-state 810 nm semiconductor diode laser as the preferred type of laser due to favorable uptake by melanin in ciliary epithelium, efficiency, lower cost, and portability [19]. The two most common variants of TSCPC are CW-TSCPC and MP-TSCPC. Destructive laser energy is delivered continuously to the ciliary body to reduce aqueous humor production in CW-TSCPC. MP-TSCPC utilizes repetitive pulses of energy separated by periods of rest to achieve more targeted treatment of pigmented tissue in the ciliary processes and minimize collateral damage to neighboring tissues [10]. CW-TSCPC is effective in reducing IOP and medication burden in refractory glaucoma cases. About 63-89% of patients who underwent CW-TSCPC achieved a target IOP of less than 22 mmHg with an absolute mean IOP reduction range of 10-23 mmHg [20]. CW-TSCPC reduces patients’ topical and systemic glaucoma medication use with approximately 1.2 average medication reduction, with 55-80% of patients who required oral acetazolamide coming off the medication after treatment [20].

The great efficacy associated with CW-TSCPC comes at a risk of severe complications, including hypotony, prolonged uveitis, damage of corneal nerves, eye pain, and phthisis. MP-TSCPC is safer and associated with fewer complications but is less potent than CW-TSCPC in terms of IOP reduction; 67-93% of patients who underwent MP-TSCPC achieved ≥20% IOP reduction from baseline at 3-12 months with a wide absolute mean IOP reduction range of 6-17 mmHg [20]. Average medication reduction at one year ranged from 0.5 to 1.6 medications across multiple studies, with 26-100% of patients who required oral acetazolamide coming off the medication after treatment [20]. The ideal laser power, duration of treatment, and probe motion are controversial for MP-TSCPC. Augmented MP-TSCPC has been described by Nirappel et al., and it involves a combination of the “sweeping” micropulse technique and the “stop-and-continue” micropulse technique with higher mean laser power and treatment duration than the manufacturer-proposed (“traditional”) settings. Augmented MP-TSCPC provided significantly more sustainable IOP control and medication reduction over traditional MP-TSCPC without compromising safety. The modality used for our patients combines the augmented MP-TSCPC and a limited application of CW-TSCPC [9]. A study by Gupta et al. describing the outcomes of this combination technique found a 16.5 mmHg and 17.9 mmHg mean drop in IOP at 12 and 18 months, respectively. The average medication burden decreased from 3.8 to 1.7 at 18 months, and no long-term sight-threatening complications were observed [10]. This technique thus limits the side effects related to CW-TSCPC while maintaining a satisfactory efficacy.

Cataract surgery in advanced glaucoma patients

Cataract surgery in glaucoma patients is the subject of ongoing research. Glaucomatous eyes are at an increased risk for complications and have more modest outcomes after cataract surgery than non-glaucomatous eyes [5]. Posterior capsular tears with vitrectomy and sulcus intraocular lens placement during cataract surgery were significantly more common in glaucomatous eyes [5]. Multiple studies have supported the pressure-lowering effect of cataract surgery in patients with and without glaucoma [21-24]. A report by the American Academy of Ophthalmology showed that phacoemulsification resulted in IOP reduction and decreased medication burden in patients with pseudoexfoliation glaucoma, primary OAG, and primary angle-closure glaucoma [25]. However, some glaucoma patients may have devastating outcomes after cataract surgery, such as reduced visual function, elevated IOP, and increased medication requirements [26-28]. Significantly higher rates of postoperative inflammation, IOP spike, and prolonged IOP elevation were observed in glaucomatous eyes undergoing cataract surgery than in non-glaucomatous eyes [5,29]. It is thus understandable why many surgeons decide to forgo cataract surgery and continue conservative treatment.

The decision and timing of cataract surgery depend on the severity and stability of the patient’s glaucoma. A positive correlation exists between glaucoma severity and the risk of complications during any ocular surgery. The added risks in managing patients with advanced glaucoma render the decision to perform cataract extraction even more delicate. In cases with refractory glaucoma, whether to delay cataract surgery or combine it with the armamentarium of possible IOP-lowering procedures is still a subject of heavy debate. Standalone trabeculectomy in phakic eyes can accelerate cataract formation. In contrast, concomitant cataract surgery in patients with moderate and severe glaucoma undergoing trabeculectomy can impact the procedure’s success and IOP-lowering effect [30,31]. Evidence is still lacking on the optimal timing to perform cataract surgery. There is still no consensus on whether combined surgery confers better outcomes regarding IOP control and complication risk than cataract surgery alone [28]. A recent Intelligent Research in Sight (IRIS®) Registry-based study by Ciociola et al. found 4.1% and 2.5% higher trabeculectomy and GDD insertion reoperation rates, respectively (P < 0.001) in standalone procedure than when combined with cataract surgery [32]. Despite all these challenges glaucoma patients still experience significant improvement in vision-related outcomes after cataract extraction [5]. Therefore, a patient-tailored assessment of the potential risks and benefits of cataract extraction and the possibility of performing a combined procedure should be made.

Cataract surgery and vision-related quality of life in glaucoma patients 

Vision loss associated with glaucoma and cataracts has a detrimental effect on a patient’s quality of life. A study by Vu et al. measured the impact of vision loss on the quality of life of 3271 participants over five years, with vision loss defined as either visual field loss or presenting visual acuity of less than 20/40. They found that both unilateral and bilateral vision loss were significantly associated with an increased probability of having problems in visual functions (i.e., reading the telephone book, watching television, and seeing faces) [33]. Non-correctable unilateral vision loss was associated with issues of safety and independent living, while non-correctable bilateral vision loss was associated with nursing home placement, lower emotional well-being, and increased use of community services [33]. Most authorities agree that cataracts can impact the vision-related quality of life in glaucoma patients [34,35]. Cataract removal has consistently improved glaucoma patients’ lives, including general vision, mobility, psychological adjustment, reading, and other ADLs [36]. Even high-risk glaucoma patients had significantly improved mean logarithm of the minimum angle of resolution (logMAR) BCVA and mean visual function questionnaire composite scores after cataract surgery [5]. A study by Lee et al. revealed that cataract extraction was associated with a significantly reduced risk of dementia when other risk factors were controlled [37]. Ocular comorbidities such as cataracts may complicate but do not necessarily preclude appropriate interventions from improving glaucoma patients’ vision-related quality of life. 

Overlooked drivers of treatment success and patient satisfaction

Quality communication and clearly defined expectation-outcome discrepancy are drivers of treatment success and patient satisfaction. Discussing potential visual outcomes before surgery limits patient expectations and enhances satisfaction. Controlling expectations in patients with concomitant cataracts and glaucoma, such as our case, is even more critical due to the added risk of complications in this population. Language is yet another substantial contributor to the quality of the patient-physician encounter. Providers are often faced with patients who do not necessarily speak the same language. Despite recent advancements in linguistic services, such patients still have difficulty communicating symptoms and understanding physician instructions. Poor quality communication between patients with limited English proficiency in anglophone countries and their providers has resulted in decreased treatment adherence, reduced patient satisfaction, and worse clinical outcomes [38]. The patient discussed in this case only used Haitian Creole and French, and her provider was coincidentally also fluent in French, thus obviating the need for an interpreter. This added advantage aided in building a trusting patient-physician relationship and allowed for better patient involvement in her treatment plan.

## Conclusions

This case illustrates the potential for visual improvement in an advanced glaucoma patient after a period of controlled IOP and the removal of a matured cataract despite limited prior expectations. Patients with “severe” or “end-stage” ophthalmic disease still necessitate thorough workup, prompt treatment, and close monitoring. Expectation management and risk-benefit calculus are critical before any decision for surgery. Ocular comorbidities complicate but do not necessarily preclude appropriate interventions that may improve patients' vision-related quality of life.
